# Chemerin Suppresses Breast Cancer Growth by Recruiting Immune Effector Cells Into the Tumor Microenvironment

**DOI:** 10.3389/fimmu.2019.00983

**Published:** 2019-05-08

**Authors:** Russell K. Pachynski, Ping Wang, Nicole Salazar, Yayue Zheng, Leona Nease, Jesse Rosalez, Weng-In Leong, Gurpal Virdi, Keith Rennier, Woo Jae Shin, Viet Nguyen, Eugene C. Butcher, Brian A. Zabel

**Affiliations:** ^1^Division of Oncology, Department of Medicine, Center for Human Immunology and Immunotherapy Programs, Washington University School of Medicine, St. Louis, MO, United States; ^2^Department of Research and Development, Palo Alto Veterans Institute for Research, Palo Alto, CA, United States; ^3^Department of Pathology, Stanford University, Stanford, CA, United States; ^4^Department of Biology, San Francisco State University, San Francisco, CA, United States; ^5^Department of Industrial and Systems Engineering, San José State University, San José, CA, United States; ^6^Dynavax Technologies, Berkeley, CA, United States; ^7^Laboratory of Immunology and Vascular Biology, VA Palo Alto Health Care Systems, Palo Alto, CA, United States

**Keywords:** chemerin, *RARRES2*, breast cancer, leukocyte trafficking, immunotherapy, NK cells, T cells

## Abstract

Infiltration of immune cells into the tumor microenvironment (TME) can regulate growth and survival of neoplastic cells, impacting tumorigenesis and tumor progression. Correlations between the number of effector immune cells present in a tumor and clinical outcomes in many human tumors, including breast, have been widely described. Current immunotherapies utilizing checkpoint inhibitors or co-stimulatory molecule agonists aim to activate effector immune cells. However, tumors often lack adequate effector cell numbers within the TME, resulting in suboptimal responses to these agents. Chemerin (*RARRES2*) is a leukocyte chemoattractant widely expressed in many tissues and is known to recruit innate leukocytes. CMKLR1 is a chemotactic cellular receptor for chemerin and is expressed on subsets of dendritic cells, NK cells, and macrophages. We have previously shown that chemerin acts as a tumor suppressive cytokine in mouse melanoma models by recruiting innate immune defenses into the TME. Chemerin/*RARRES2* is down-regulated in many tumors, including breast, compared to normal tissue counterparts. Here, using a syngeneic orthotopic EMT6 breast carcinoma model, we show that forced overexpression of chemerin by tumor cells results in significant recruitment of NK cells and T cells within the TME. While chemerin secretion by EMT6 cells did not alter their phenotypic behavior *in vitro*, it did significantly suppress tumor growth *in vivo*. To define the cellular effectors required for this anti-tumor phenotype, we depleted NK cells or CD8+ T cells and found that either cell type is required for chemerin-dependent suppression of EMT6 tumor growth. Finally, we show significantly reduced levels of *RARRES2* mRNA in human breast cancer samples compared to matched normal tissues. Thus, for the first time we have shown that increasing chemerin expression within the breast carcinoma TME can suppress growth by recruitment of NK and T cells, thereby supporting this approach as a promising immunotherapeutic strategy.

## Introduction

Breast cancer is one of the most common malignancies with an estimated ~266,000 new cases in 2018, according to SEER estimates. The impact of infiltrating lymphocytes on breast cancer patient outcomes has been studied in several contexts, but in general is a favorable prognostic factor (1–3). The presence of pre-existing immune effectors cells within the tumor microenvironment (TME) within breast and other tumor types can not only predict response to traditional cytotoxic chemotherapy, but also immunotherapies (4–6). Compared with tumor types that are more responsive to checkpoint immunotherapy, however, there is a relative paucity of infiltrating lymphocytes in breast cancer (7). Thus, strategies to enhance recruitment of immune effector cells to the breast TME are highly desirable.

Chemerin (retinoic acid receptor responder 2; *RARRES2*) is a leukocyte chemoattractant initially discovered as being highly up-regulated in the skin by the synthetic retinoid tazarotene (8). Chemerin is widely expressed throughout tissues and has myriad roles including the chemoattraction of innate cells [e.g., NK cells, macrophages, dendritic cells (DCs)] (9–11), functioning as an important antimicrobial agent in the skin (12), and is able to induce angiogenesis in human endothelial cells (13), suggesting chemerin may be a key factor in early immune responses to infection, injury, and/or inflammation. Chemerin is initially secreted in an inactive pro-form, prochemerin, which is then cleaved by specific proteases to become bioactive (9, 10). Chemerin has three described serpentine cell membrane receptors: chemokine-like receptor 1 (CMKLR1; ChemR23), C-C chemokine receptor-like 2 (CCRL2), and G protein-coupled receptor 1 (GPR1) (9, 14, 15). CMKLR1 is a chemotactic cellular receptor, while, atypical chemoattractant receptor CCRL2 likely acts to sequester and concentrate chemerin at sites of CCRL2 expression, such as on activated endothelial cells (14–17). The function of GPR1 is poorly understood, though it is reported to be expressed in the CNS (18, 19).

Chemerin/*RARRES2* has been studied in the context of several different tumor types, with its dysregulation dependent on the specific context. While we and others have reported on several tumor types where chemerin/*RARRES2* is significantly down-regulated compared to normal tissue counterparts (e.g., melanoma, lung, prostate, liver, adrenal, etc.) (20–25), chemerin/*RARRES2* has been shown to be up-regulated in fewer tumor types (e.g., mesothelioma, squamous oral cancers) (26–28). Several groups have correlated chemerin/*RARRES2* expression levels in the TME with clinical outcomes, showing improved patient survival in those patients with higher expression levels (20–22, 24). Importantly, two of these studies also evaluated the tumor biopsies for infiltrating leukocytes, showing an increase and correlation between higher chemerin levels and infiltrating NK cells in those patients with improved overall survival (20, 21).

Our group was the first to show that in a mouse melanoma model, overexpression and secretion of chemerin protein by tumor cells increased total CD45+ tumor infiltrating leukocytes (TIL), resulting in significantly suppressed tumor growth. In this model, the effect was mediated by NK cells, as depletion via anti-asialo GM1 resulted in complete abrogation of chemerin's tumor suppressive effects (22). In contrast, T cells were dispensable, as RAG deficiency had no effect on the anti-melanoma effects of chemerin *in vivo* (22). Importantly, neither engineered chemerin expression nor incubation of mouse B16F0 melanoma cells with exogenous, recombinant chemerin affected *in vitro* growth or phenotype, suggesting chemerin's main anti-tumor activity was due primarily to its ability to recruit immune effector cells into the TME.

Here, we studied the effect of chemerin/*RARRES2* overexpression using the transplantable orthotopic syngeneic EMT6 breast carcinoma model, which has been shown to be responsive to immunomodulation in a variety of settings (29–31). Utilizing a similar approach as in the B16 model, we engineered EMT6 tumor cells to express and secrete functional chemerin within the TME and then assessed the impact on tumor growth and TIL. Chemerin overexpression significantly suppressed tumor growth, which correlated with an increase in TIL. Depletion studies identified NK and CD8+ T cells as key effector leukocytes mediating chemerin's anti-tumor activity, suggesting an interplay between innate and adaptive arms. In human breast tissue, chemerin/*RARRES2* RNA expression was significantly reduced in malignant samples compared to normal controls. Taken together, these data suggest that loss of chemerin/*RARRES2* expression occurs in breast cancer during tumorigenesis, potentially as an immune evasion mechanism, and that restoring or enhancing chemerin levels within the TME may prove efficacious in increasing TIL, thereby slowing or reversing tumor progression in the clinic.

## Materials and Methods

### Microarray Analysis

Publicly available breast cancer studies were evaluated using the Oncomine database (www.oncomine.org), in which expression data has been curated using statistical methods and standardized normalization technique as previously described (32). The two largest breast cancer studies comparing normal to malignant tissues were selected: Curtis et al. (http://www.ebi.ac.uk/ega/studies/EGAS00000000083) (33) and TCGA (http://tcga-data.nci.nih.gov/tcga) (34). The Curtis dataset contains 1,992 breast carcinoma samples and 144 paired normal breast samples which were analyzed for the METABRIC project using the Illumina HumanHT-12 V3.0 R2 Array. The TCGA data included 532 invasive breast carcinomas and 61 paired normal breast tissue samples using level 2 (processed) data from the TCGA portal. The *RARRES2* probe was selected for normal, invasive/infiltrating ductal carcinoma (IDC) and invasive/infiltrating lobular carcinoma (ILC) subsets, and gene expression (mRNA) data were shown as log2 transformed, median centered per array with *p*-values and fold change between subsets generate by Oncomine.

### Mice and Cell Lines

All mice were used in experiments were purchased from The Jackson Laboratory. Wild type or Rag1 knockout (RAG KO; #003145, *Rag1*^*tm*1*Mom*^) (35) female BALB/c mice were used as indicated. Mice were maintained in the facilities at Washington University under the direction and guidelines of the Division of Comparative Medicine and used at approximately 9–12 weeks of age. All animal experiments were conducted in accordance with approved Washington University and National Institutes of Health Institutional Animal Care and Use Committee guidelines under an approved protocol (#20140232). The EMT6 mouse mammary carcinoma cell line was purchased from ATCC (CRL-2755). L1.2 cells transfected to express mouse CMKLR1 were a kind gift from BA Zabel. Cell lines were grown in complete media consisting of RPMI 1640 or DMEM supplemented with 10% FBS, sodium pyruvate, penicillin/streptomycin, and beta-mercaptoethanol, with or without appropriate antibiotics for selection. EMT6 cell lines (wild type and transduced) were serially tested for mycoplasma and found to be negative using the MycoProbe Mycoplasma Detection Kit (R&D Systems).

### EMT6 Clone Production

The full-length gene that encodes mouse active chemerin, mouse *RARRES2*, was inserted into the lentiviral transfer vector pCDH1-MSC1-EF1-Puro (System Biosciences) using the NheI and EcoRI restriction enzyme digestion sites. Empty vector pCDH1-MSC1-EF1-Puro was used to produce control lentivirus. 293T/17 cells were grown in DMEM complete media in 10 cm dishes for 16 h before transfected with packaging plasmid (Δ8.2), coat protein vector (pCMV-VSV-G) and transfer vector (pCDH-Puro-wt *RARRES2* or pCDH-Puro Empty vector) by using the FuGENE® HD Transfection Reagent (Promega) according to the manufacturer's protocol. The culture supernatants containing lentiviruses were collected at 48 and 72 h post transfection. The collected media were centrifuged at 300 × g to remove cell debris and followed by filtration with 0.45 μM filters. Viral supernatants were either used immediately for cell transduction or stored at −80°C. To create EMT6 cell lines with constitutive chemerin expression or control vector, viral supernatants added with polybrene were used to infect wild type EMT6 cells. Starting 24 h infection, cells were selected with media containing 2 μg/ml puromycin for 3 days. Culture media containing puromycin was replaced daily. Monoclonal cell populations were obtained by limiting dilution.

### *In vitro* Cell Line Evaluation

EMT6-pCDH-VEC or EMT6-pCDH-*RARRES2* cells (1,000 cells/well) were plated in 96-well black walled plates (Corning). Cells were grown in a 5% CO_2_ humidified incubator at 37°C for the indicated days. On each day, alamar blue reagent (ThermoFisher Scientific) was added directly to each well, the plates were incubated at 37°C for 1–4 h and the fluorescence signal was measured according to the manufacturer's protocol. Data were shown as relative fluorescence values compared with that of day 0, which was normalized to 1. Control and chemerin-expressing EMT6 lines were plated at 200 k/ml/well in 24 well plates and evaluated for chemerin secretion by using a mouse chemerin ELISA (R&D Systems) on 48 h conditioned media. Surface marker expression of control and chemerin-expressing clones was evaluated by flow cytometry with indicated monoclonal antibodies and appropriate isotype controls (Biolegend). The functionality of secreted chemerin was tested using conditioned media from control and chemerin-expressing clones in chemotaxis assays. Briefly, 96 well HTS Transwell Permeable Supports with 5 μm pores (Corning) were used according the manufacturers protocol; 250 k mCMKRL1+ L1.2 cells/75 μl were placed in the top chamber and 240 μl of complete media +/− 3 nM recombinant, active mouse chemerin (R&D Systems), or conditioned media in the bottom chamber. Assays were left at 37°C for ~1–1.5 h. Migrated cells in the bottom chamber were counted and percent migration calculated.

### Tumor Inoculation

To evaluate the effect of constitutive chemerin secretion on *in vivo* tumor growth, control or chemerin-expressing EMT6 breast tumor cells (0.5–1 × 10^6^) were inoculated subcutaneously into 9–12 weeks old female BALB/c mice (JAX). Prior to inoculation, EMT6 lines were grown to ~60–80% confluence to ensure log-growth kinetics, and viability was tested using trypan blue and ensured to be ~>95% (or cells were not used). Tumor growth was measured every 2–4 d by calipers, and size was expressed either as the volume product of perpendicular length by width in square millimeters, or by tumor size as indicated by width × length (in square mm). Mice were euthanized when tumor size reached ~400 mm^2^ or when tumor sites ulcerated or at indicated time points for TIL analyses.

### *In vivo* Leukocyte Depletion

Mice were injected i.p. with 100 μl of anti-asialo GM1 or control rabbit sera (Wako Chemicals) diluted 1:10 in PBS. Mice were treated with antibodies on day 1, day 0 and every 2–3 days after tumor inoculation. NK depletion efficiency was determined by staining blood cells collected from the venous sinus. Briefly, blood samples were isolated via retro-orbital bleed and washed once with PBS. After centrifugation at 300 × g for 5 min, cells were stained with CD45, CD3, and DX5 or its isotype control (Biolegend) and analyzed by FACS. For CD4+, CD8+ T cell depletion, mice were injected i.p. with 250 μg/500 μl PBS of anti-CD4 (clone GK1.5, BioXCell), anti-CD8β (Lyt 3.2) (clone 53-5.8, BioXCell) or both, and rat IgG (Sigma) for control. Antibodies were given weekly for 3 doses. Depletion efficiency was determined by staining blood cells collected via retro-orbital bleed with CD45, CD3, CD4, and CD8 antibodies (Biolegend) and analyzed by FACS.

### Evaluation of Tumor Infiltrating Leukocytes

At indicated time points, whole subcutaneous tumors were resected en bloc including overlying skin and subcutaneous tissues. Tumors were then processed into single cell suspensions as previously described (22). Briefly, cells were counted using trypan blue, and samples were blocked with PBS/FBS containing 1% rat serum and Fc block (anti-CD16/32; Biolegend). Stained samples were analyzed on a BD Fortessa. For live/dead cell discrimination, AmCyan LIVE/DEAD Fixable Dead Cell Stain kit (Invitrogen) was used. Antibodies or appropriate isotype controls were purchased from Biolegend and FlowJo software (Tree Star) was used for analysis, with gating based on appropriate isotype control staining, and percentages expressed as shown of total live tumor cells or total live CD45+ cells, as indicated. FACS analyses was used to define the follow leukocyte subsets (all Live+CD45+): plasmacytoid DCs (pDCs; Lin-CD11c^int^B220^hi^), conventional DCs (cDCs; Lin- CD11c^hi^B220^low^), CD4 (CD3^+^CD4^+^) T cells, CD8 (CD3^+^CD8a^+^) T cells, total T cells (CD3^+^CD4^+^CD8^+^), NK cells (CD3-DX5+), monocyte/macrophages (Lin-CD11b^+^GR1^−^), MDSCs (Lin-CD11b^+^GR1^+^), M1 (Lin-CD11c^hi^F4/80+), and M2 (Lin-CD11c^low^F4/80+) macrophages, CD19^+^ B cells (CD3-CD19^+^). CD8+ T cell subsets were based on staining with CD44 and CD62L: naïve (CD44^low^CD62L^hi^), effector (CD44^int^CD62L^low^), or memory (CD44^hi^CD62L^low^).

### Breast Tissue Microarrays

Tissue microarray (TMA) Breast Tissue FFPE sections were collected from the St. Louis Breast Tissue Registry (funded by The Department of Surgery at Washington University School of Medicine, St. Louis, MO) under IRB-approved institutional protocols. All patient information was de-identified prior to sharing with investigators. Data and tissue was obtained in accordance with the guidelines established by the Washington University Institutional Review Board (IRB #201102394) and WAIVER of Elements of Consent per 45 CFR 46.116 (d). Each TMA core was 5 μm thick and 2 mm in diameter. Normal and Tumor tissue was confirmed by a Board-Certified Pathologist (Dr. Marshall Poger) using a stained Hematoxylin and Eosin (H&E) section. Breast Tumor TMA section contained 37 IDC cases and 8 ILC cases. Normal TMA Section contained 45 cases of Terminal Ductal-Lobular Unit (TDLU) with 1 Tonsil and 4 Liver cores for control and TMA positioning.

### Quantitative Real-Time PCR

De-identified, paired RNA samples of malignant or non-malignant human breast tissues were from the Siteman Cancer Center Tissue Procurement Core, collected under an IRB-approved research protocol (#201106191). Quantitative Real-Time PCR was carried out using the SYBR® Green master mix (Bio-Rad) with the real-time PCR primers for human chemerin and the housekeeping gene GAPDH (sequence listed below). Measurements were standardized to GAPDH using delta-delta Ct methods. RNA from human liver was the positive control for chemerin expression. RNA from RAJI cells was the negative control. Data were expressed as fold expression levels of negative control (RAJI, normalized to 1). Data shown are mean ± SEM of two independent experiments using identical starting RNA. Significant outliers identified by Grubbs' test were removed. The primers used for human *RARRES2* have been previously described (36): Forward: 5′- TGGAAGAAACCCGAGTGCAAA-3′; Reverse: 5′-AGAACTTGGGTCTCTATGGGG-3′ Primers for human GAPDH: Forward: 5′- GAGTCAACGGTTTGGTCGTATTG-3′; Reverse: 5′- ATGTAGTTGAGGTCAATGAAGGGG-3′.

### *In situ* Hybridization and Analysis

Manual chromogenic RNAScope (ACDBio) was performed using company protocols on TMA tissue sections to detect target RNA at single cell level. Tissue pre-treatment (Liver) included baking for 1 h at 60 degrees Celsius, deparaffinization using xylene and alcohol, RNAscope® Hydrogen Peroxide (ACD# 322335) treatment for 10 min at RT and protease treatment (RNAscope® Protease Plus ACD# 322331) for 30 min at 40 degrees Celsius using the HybEZ Oven. Pre-treatment of non-adherent cells (RAJI) included fixation by 10% NBF and dehydration in series of 50, 70, and 100% ethanol. Cells were treated with RNAscope® Hydrogen Peroxide for 10 min at RT (ACD# 322335) and treated with RNAscope® Protease III (ACD# 322337) for 30 min in 40 degrees Celsius using HybEZ oven. For all tissue sections and non-adherent cells, ACDBio pre-treatment protocol was used according to manufacturer's instructions. Detection of specific probe binding sites was with RNAScope 2.5 HD Reagent kit—brown from ACD (Cat. No. 322310). Single ISH detection for human *RARRES2* (ACD Probe: 457921), Positive Control Probe (PPIB - ACD Probe: 313901) and Negative Control Probe (Dapb—ACD Probe: 310043) was performed manually using RNAscope® 2.5 HD Reagent Kit-Brown (ACD, 322310). Target probes were hybridized for 2 h at 40 degrees Celsius using HybEZ oven and a series of 6 amplification steps followed. A DAB-based chromogenic reagent was used to detect the brown signal for the *RARRES2* probe expression. The experimental procedure followed the manufacturer's instructions for single plex assay. Positive staining was indicated by brown granular dots present in the nucleus and/or cytoplasm.

Quantitative analysis was completed using regions of interest (ROIs) and by random sampling. The ROIs for Normal and Tumor breast tissue were manually selected by a Board-Certified Pathologist (Dr. Marshall Poger) for imaging. Random sampling was done by numbering each core on the TMA section and using a random number generator to select which TMA core was to be selected for analysis. HALO Software by Indica Labs was used, specifically with the RNAScope ISH Module per recommendation by ACD, with user-defined thresholds. This module allowed the user to teach HALO software to recognize hematoxylin (blue) and positive signal (brown granular dots). Positive signal is reported by number of RNA copies. The Cytonuclear Module was used to teach HALO Software to recognize hematoxylin (blue) to identify nuclei. This generated a contrasted image allowing the user to count the number of nuclei in the region of interest. ISH module provided the user the number of RNA copies and the Cytonuclear module provided the user the number of cells. Thus, RNA copies per nuclei was determined allowing analysis to be normalized to each nuclei. Slides were imaged using a Nikon eclipse 50i microscope at 40x resolution. Three comparable regions of interest for tumor (IDC and ILC) and normal breast (TDLU) were subject to HALO Software for image analysis.

### Statistical Analysis

*In vitro* and *in vivo* tumor data was plotted using Prism software v7 and further analyzed with InStat (GraphPad Software). Differences between groups were evaluated by applying unpaired Student's *t*-test or non-parametric Mann-Whitney test, as indicated. Paired human RNA samples were evaluated by a paired student's *t*-test. *p* < 0.05 were considered significant.

## Results

### Reduced *RARRES2* Expression in Human Tumors and Associated Poor Survival Outcome

**Figure 1:**
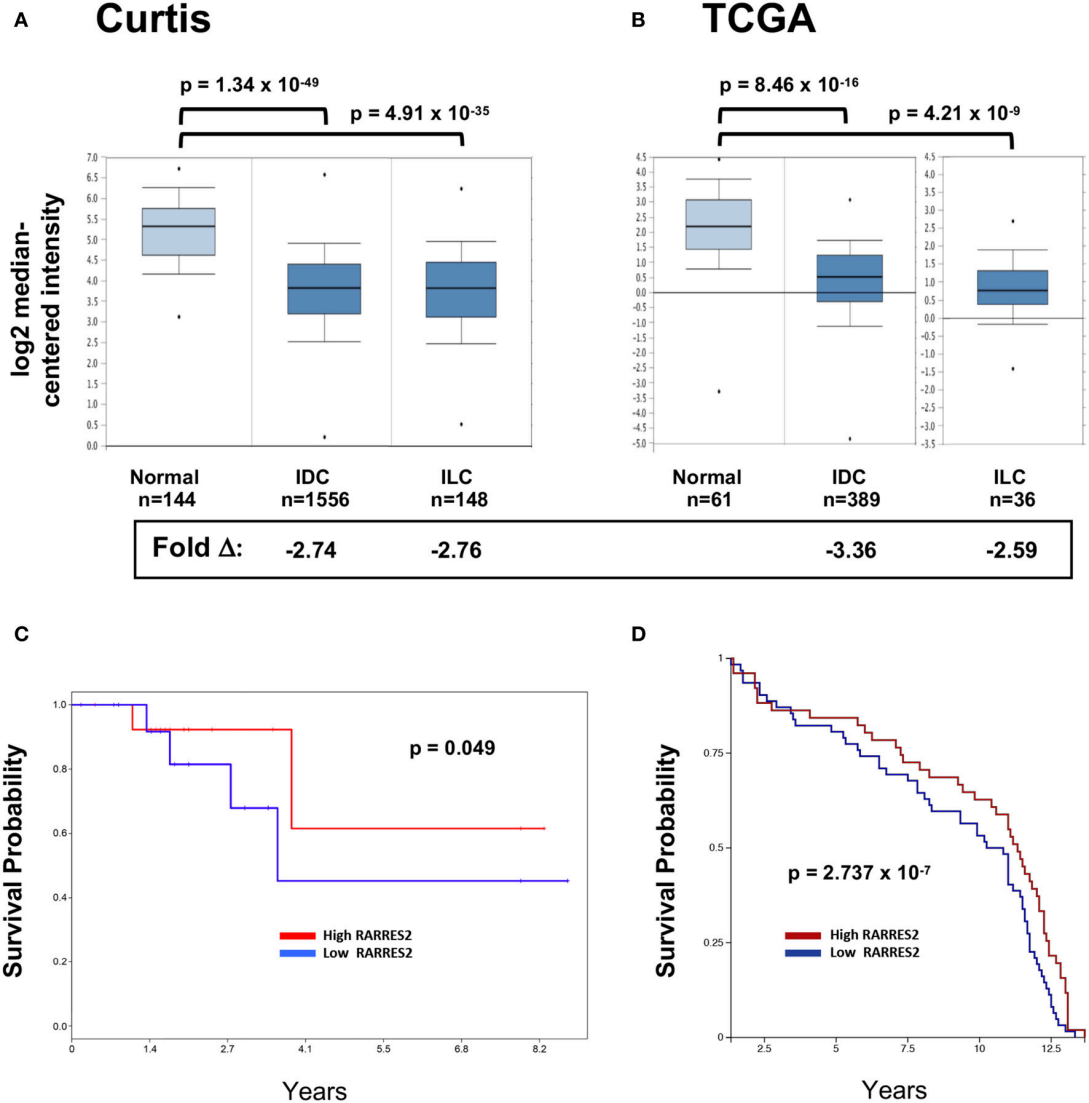
*RARRES2* microarray expression in breast tissues. The two largest mRNA studies comparing normal and malignant breast tissues were selected in Oncomine (www.oncomine.org) for analysis. **(A)** Curtis, *n* = 2,136 total, **(B)** TCGA, *n* = 593 total. Both infiltrating ductal carcinoma (IDC) and infiltrating lobular carcinoma (ILC) subsets show significantly lower expression of *RARRES2* mRNA when compared to normal breast tissue. *RARRES2* probes were selected and relative expression by log2 median-centered intensity plotted for normal, IDC, and ILC subsets within each study. Oncomine calculated *p*-values and fold change compared with normal subset are shown. Down-regulation of chemerin in breast cancer can be associated with poor survival outcomes. **(C)** mRNA microarray data (accession # GSE6130-GPL887) from a cohort of 33 patients with breast cancer. mRNA microarray data was visualized using PROGgeneV2. The patients were stratified according to chemerin expression (divided at 50th percentile), and survival plotted for each group. Hazard ratio: 0.42 (0.18–1.00), *p*-value: 0.049, indicating that low chemerin levels significantly correlated with poor survival in this group. **(D)** mRNA microarray data (Caldas, Naderi Gene Exp 2007) from a cohort of 135 early-stage breast cancer, visualized using the UCSC Xena Browser. The patients were stratified according to chemerin expression (divided at 50th percentile), and survival plotted for each group. *p*-value: 2.737 × 10^−7^, indicating that low chemerin levels significantly correlated with poor survival in this group.

**Figure 2:**
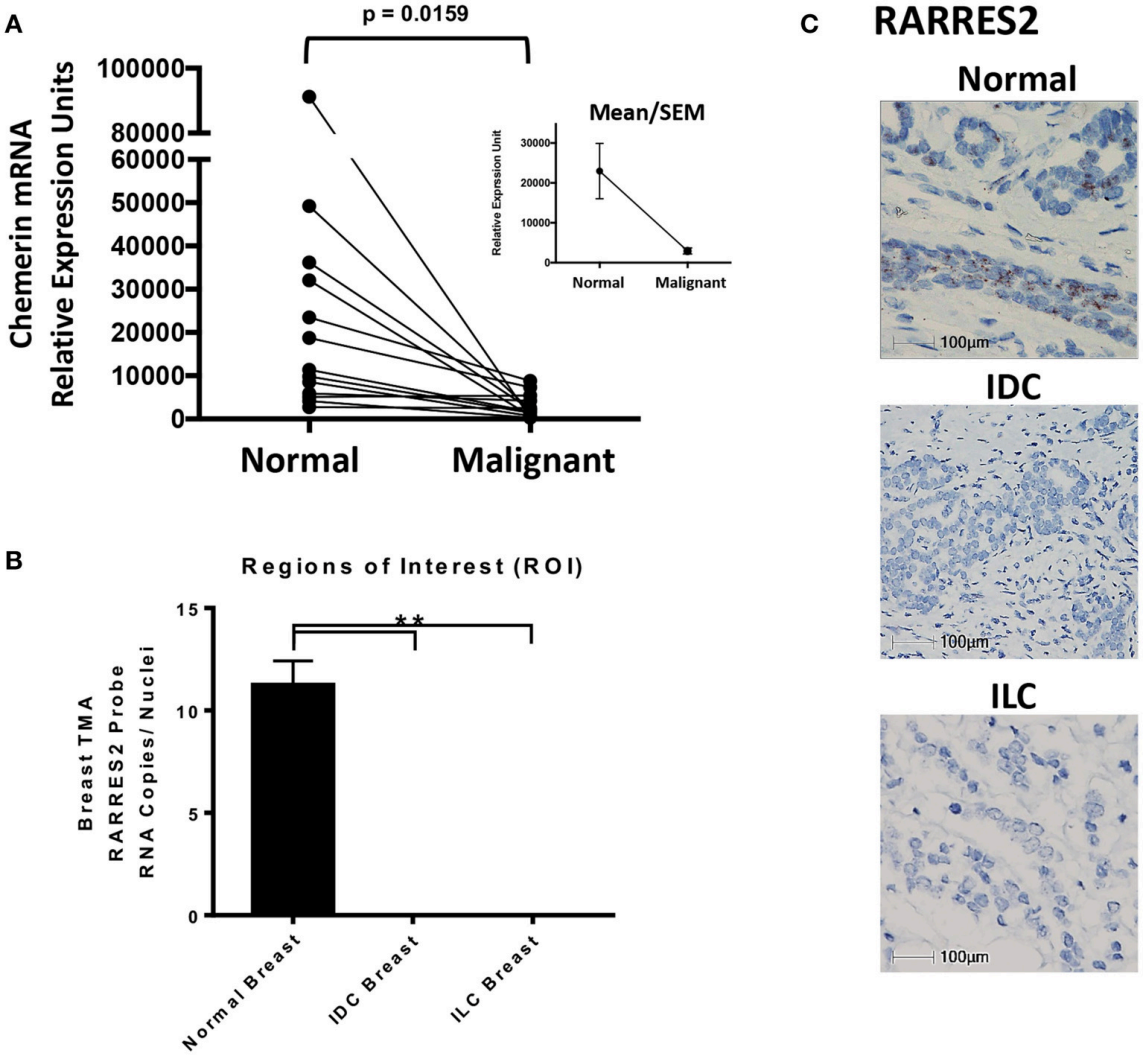
Chemerin RNA expression in human breast tissue. **(A)** Real-time quantitative PCR (RT-qPCR) of chemerin mRNA expression in matched normal and malignant breast tissue. RNA was isolated from paraffin embedded tissues and assessed for concentration, purity, and integrity. Chemerin expression was normalized to GAPDH loading control for each sample (^**^p = 0.0159 compared to normal breast tissue, *n* = 13 for each subset using two tailed paired *t*-test). Inset shows group means/SEM. **(B)** Quantified *RARRES2* RNA expression in normal breast, IDC breast, and ILC breast tumor tissue microarrays (TMA) using RNAScope *in-situ* hybridization (ISH). Three comparable regions of interest (ROI) from each case of normal breast (*n* = 7) or tumor (IDC and ILC; *n* = 7 each) were subject to HALO Software for image analysis. The ROIs for normal and tumor breast tissue were manually selected for imaging/analysis. RNA expression is normalized to the number of nuclei in each image to determine RNA copies per nuclei. (^**^p = 0.0001 compared to normal breast using a one sample *t*-test). Results are representative of two TMAs containing (1) 45 normal cases and (2) 37 IDC cases and 8 ILC cases. **(C)** Representative ISH images for *RARRES2* RNA expression in normal, IDC breast, and ILC breast tissue. Slides were imaged using a Nikon eclipse 50i microscope at 40X resolution; 100 mm bar shown. Positive staining is indicated by brown granular dots present in the cell nucleus and/or cytoplasm.

We and others previously showed that chemerin/*RARRES2* expression is commonly down-regulated in multiple tumor types, including breast cancer, compared to normal tissue controls *RARRES2* (22). Our published expression analysis was limited to the publicly available GEO microarray datasets, thus, to confirm reproducibility we sought to further investigate chemerin expression in larger datasets. Here, we analyzed the two largest breast cancer datasets with data for *RARRES2* that were curated within the Oncomine database (32). Chemerin/*RARRES2* expression in both Curtis (Figure 1A) and TCGA (Figure 1B) datasets was significantly decreased by approximately 2.6- to 3.4-fold in tumor specimens compared to normal (33, 34). Subsequently, to examine the association between reduced *RARRES2* expression and patient survival outcome, we analyzed two sets of mRNA microarray data with cohort sizes of 33 breast cancer patients and 135 early-stage breast cancer patients, respectively, and found that low chemerin levels significantly correlated with poor survival outcomes in both groups (Figures 1C,D). By *in situ* hybridization (ISH) comparing normal tissues to both invasive ductal carcinomas (IDC) as well as invasive lobular carcinoma (ILC)—the two most common histologic subtypes (37)—chemerin/*RARRES2* was also down-regulated in the tumor samples (Figure 2). Thus, across multiple datasets and analytical expression methods, chemerin/*RARRES2* was consistently down-regulated in malignant breast cancer samples vs. controls, and reduced chemerin/*RARRES2* expression was correlated with poor survival outcome.

### Reduced Chemerin Expression in Human Invasive Breast Cancers

Next, we wanted to independently validate the findings of our public microarray analyses (Figure 1). We collected human breast tissues from two different sources and, using two different modalities, evaluated chemerin expression via measurement of *RARRES2* mRNA. Matched total RNA from normal and malignant breast tissues (*n* = 13 patients with IDC) were obtained from the Siteman Cancer Center Tissue Procurement Core. De-identified frozen samples were collected under approved consents, pathologically reviewed, and processed into RNA per established protocols. RNA quantity and quality (i.e., RIN) was assured and validated primers for human *RARRES2* (36) were used in real-time quantitative PCR. Expression of *RARRES2* mRNA in malignant breast tissues was significantly reduced compared to patient matched, normal tissue (Figure 2). Group mean/SEM of individually matched samples (Figure 2A) are shown. Next, using samples collected from the St. Louis Breast Tissue Registry under IRB-approved institutional protocols, we then constructed normal (*n* = 45 cases) and malignant breast tissue microarrays (TMA) incorporating both IDC (*n* = 37 cases) and ILC (*n* = 8 cases). Utilizing ACDBio RNAscope, RNA *in situ* hybridization (ISH) was performed. *RARRES2* was undetectable in both IDC and ILC samples compared to low but significant *RARRES2* signal in normal tissues (Figure 2B). Duplicate TMA slides were used with positive and negative probes (ACDBio), in parallel with human liver (*RARRES2*-positive) and Raji cells (*RARRES2*-negative) as controls (Supplemental Figure 1). Representative images are shown in Figure 2C, with the majority of staining localized to epithelial components of the normal breast tissue. Taken together, our data confirms significant down-regulation of *RARRES2* mRNA expression in both IDC and ILC compared to normal breast tissues.

### Forced Expression of Chemerin by EMT6 Breast Carcinoma

**Figure 3:**
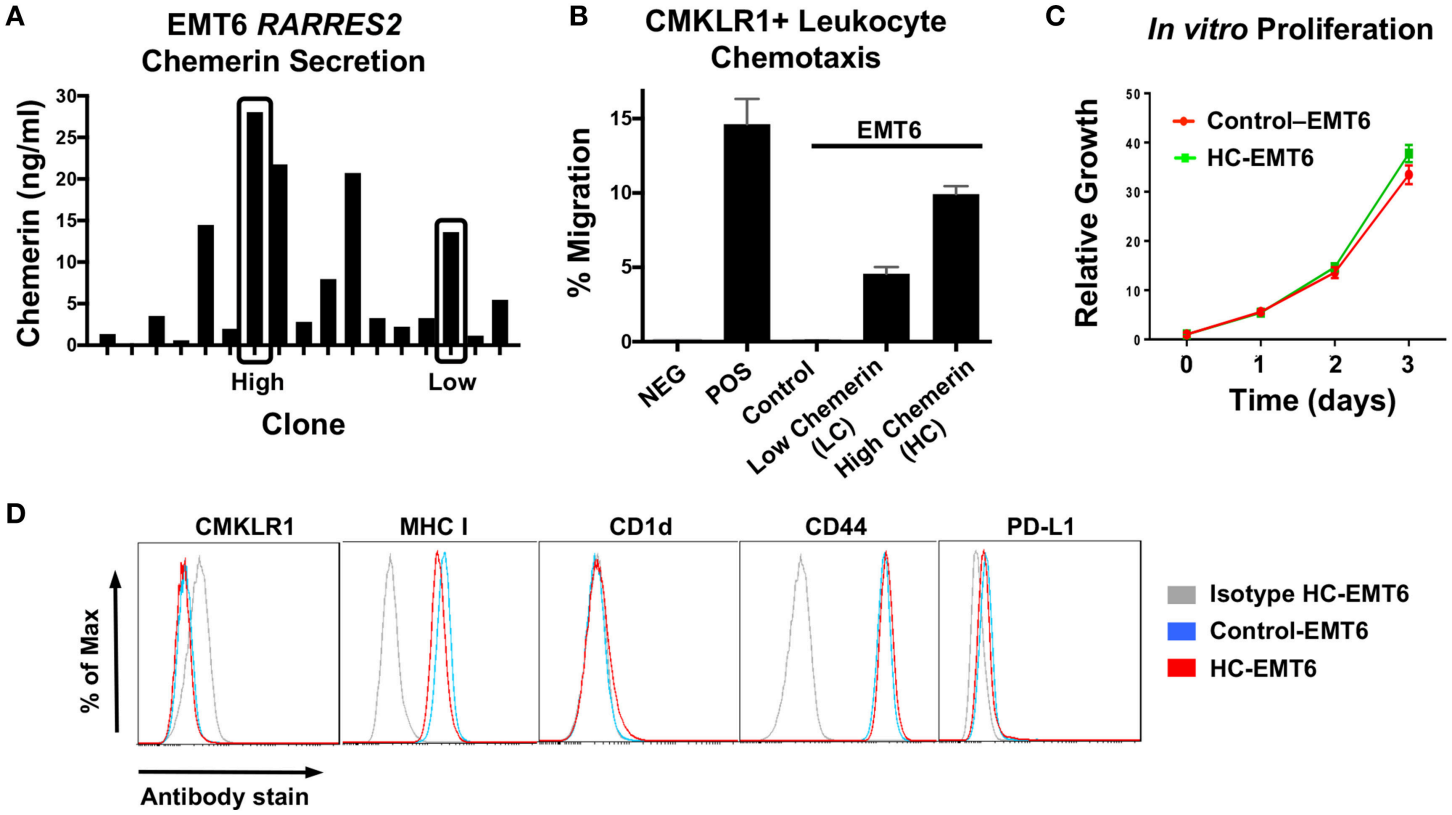
Evaluation of chemerin-expressing EMT6 tumor lines. The mouse breast carcinoma line EMT6 was used to study the impact of forced chemerin expression on tumor growth. Wild type EMT6 cells were transduced using the lentiviral transfer vector pCDH1-MSC1-EF1-Puro, with the full length mouse *RARRES2* gene inserted (*RARRES2*) or not (control). **(A)** Clonal cell lines (*n* = 17) were generated and chemerin protein secretion in clonal conditioned media samples was quantified by ELISA. Clones with low (LC) and high chemerin (HC) secretion (indicated by boxes) were selected for subsequent *in vitro* and *in vivo* analysis. **(B)** Chemotaxis assays using CMKLR1+L1.2 transfectants and 5 micron transwell chambers were performed to confirm functionality of EMT6-secreted chemerin. Conditioned media was used from control and chemerin-expressing tumor lines (LC, HC). Media alone (NEG) and 3 nM recombinant mouse chemerin (POS; R&D Systems) were used as controls. The normalized “percent of input” migration is shown; mean/SEM plotted for duplicate wells for each condition. Representative proliferation and chemotaxis assays are shown, and were each performed several times prior to tumor inoculation in mice. **(C)** Control and high chemerin-expressing lines were evaluated for *in vitro* cell proliferation using an alamar blue assay; relative fluorescence values normalized to 1 on day 0 are shown. **(D)** Surface expression of CMKLR1, MHC class I, CD-1d, CD44, and PD-L1 was determined by FACS for control and high chemerin-expressing EMT6 tumor lines. For each marker indicated, the appropriate isotype control antibody (gray) is shown.

After confirming down-regulation of *RARRES2* mRNA in additional human studies, we then set out to favorably modulate chemerin expression in the EMT6 mammary carcinoma model. The EMT6 tumor line is a clonal isolate from a mouse mammary carcinoma that arose from an implanted hyperplastic alveolar nodule (38), and has been shown to be responsive to immunomodulation (31, 39, 40). In order to test our hypothesis that forced overexpression of chemerin by tumor cells would act to recruit anti-tumor leukocytes and suppress tumor growth, we used lentiviral transduction to introduce the mouse *RARRES2* gene into EMT6 tumor cells. The pCDH1-MSC1-EF1-Puro (System Biosciences) vector was used to produce either control (empty vector) or *RARRES2* viral particles for transduction. Control and chemerin-expressing EMT6 clonal lines were generated by limiting dilution plating. Evaluation of tumor-secreted chemerin was assessed by mouse chemerin ELISA (R&D Systems). Both wild type and control-transduced EMT6 lines showed no detectable chemerin by ELISA (not shown), while *RARRES2*-transduced clones showed significant production of secreted chemerin in the ng/ml range (Figure 3A). From these, two clones, one with low (LC) and one with high (HC) chemerin expression, were then selected for further evaluation. In order to determine if the tumor-secreted chemerin was functional and active, we utilized standard chemotaxis assays using 5 um pore transwell chambers. Conditioned media from both control and chemerin-expressing tumor lines was evaluated. The mouse pre-B lymphocyte cell line L1.2 engineered to express high levels of mouse CMKLR1 (10) was used to assess chemerin-dependent migration. Conditioned media from control transduced lines was unable to induce CMKLR1+ L1.2 cell chemotaxis, while conditioned media from chemerin-expressing tumor lines triggered robust migration comparable to recombinant, active chemerin (3 nM, R&D Systems). Chemotaxis of CMKRL1+ L1.2 cells in the HC clone was ~2-fold compared to the LC clone, in line with measured secreted chemerin levels (Figure 3B). In order to assess the effects of chemerin production and secretion on *in vitro* tumor cell proliferation, we utilized an alamar blue assay (ThermoFisher) and measured growth as a function of fluorescence signal over several days. There were no consistent differences between control or chemerin-expressing EMT6 clones (Figure 3C). Next, we looked at expression of several common surface markers involved in tumor-immune recognition (MHC class I, CD1d, PD-L1) as well as tumor cell migration and invasion (CD44) (41). While CMKLR1 has been reported to be expressed on some human tumors (42), we did not see detectable surface levels of CMKLR1 above isotype control, in line with our prior studies of the mouse melanoma line B16F0 (22). Figure 3D shows comparable phenotypic expression of these markers between control and chemerin-expressing tumor lines. These data show that transduction with *RARRES2* and expression/secretion of chemerin by EMT6 tumor cells does not appear to meaningfully impact *in vitro* growth or the immunophenotype of key surface proteins, and that secreted chemerin is functionally active and can induce migration of CMKLR1+ cells.

### Chemerin Overexpression Suppresses EMT6 Tumor Growth *in vivo*

**Figure 4:**
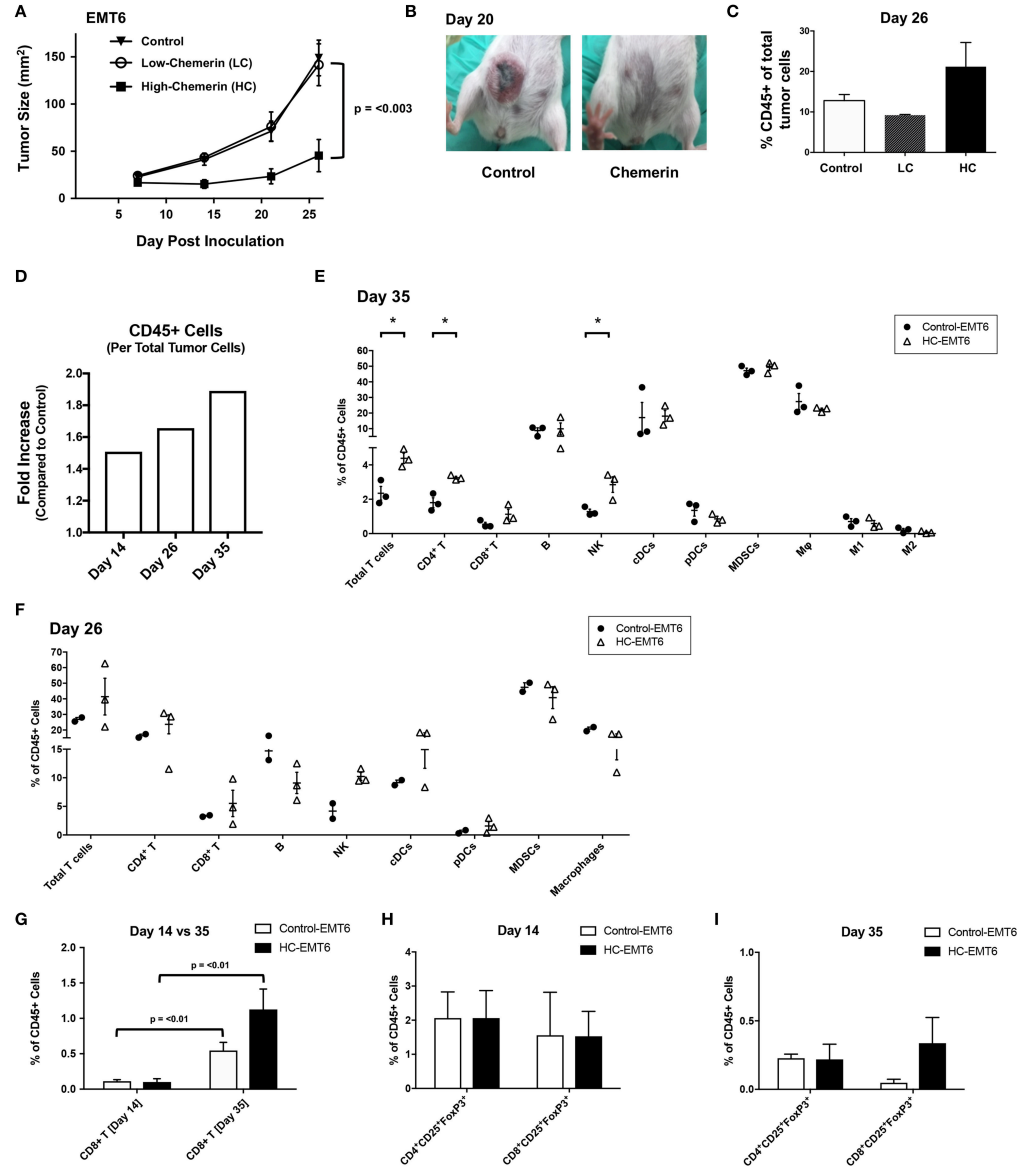
High chemerin expression by EMT6 tumors suppresses *in vivo* growth and results in altered TIL makeup. **(A)** High and low chemerin-expressing EMT6 clones were used in parallel with a control transduced EMT6 cells. 0.5–1 × 10^6^ tumor cells were inoculated subcutaneously into 9–12 weeks old female BALB/c mice. Tumor growth was serially assessed using calipers. Mean/SEM shown with groups *n* = 10 per indicated line. Two-way ANOVA with Tukey's multiple comparisons test show no significant differences between control and LC groups, with p-value as indicated for differences compared to HC group. **(B)** Representative images of mice from control and chemerin-expressing groups showing significant *in vivo* suppression of tumorigenesis, 20 days post-inoculation. **(C)** Percent CD45 positive of total tumor cells by FACS analysis within the tumor microenvironment (TME) shown for **(A)** tumors (*n* = 2–3/group) resected at time of euthanasia (day 26). **(D)** Graph of fold increase showing CD45+ cells per total tumor cells, comparing HC-EMT6 tumors to control-EMT6 tumors resected at time of euthanasia (day 14, *n* = 6/group; day 26, *n* = 2–3/group; day 35, *n* = 3/group) and analyzed via FACS. Graph depicts two independent experiments; tumors collected on day 14 and day 35 are derived from the same experiment. **(E,G–I)** In a separate cohort of mice, we euthanized animals at pre-defined timepoints for TIL analysis. Of the mice that were initially inoculated; six mice per group were euthanized on day 14 for FACS analysis, and an additional three mice per group were euthanized on day 35 for further FACS analysis. Graphs show mean/SEM values; statistical significance (defined in Methods) was determined between groups using a 2-sided unpaired *t*-test. **(E)** FACS analysis of TIL from control-EMT6 or HC-EMT6 tumors (*n* = 3/group) resected on day 35. **(F)** FACS analysis of TIL from control-EMT6 or HC-EMT6 tumors resected on day 26 (*n* = 2–3/group). **(G)** FACS analysis of TIL from HC-EMT6 tumors (*n* = 6) resected on day 14 compared to TIL from HC-EMT6 tumors resected on day 35 (*n* = 3), specifically showing the CD8+ T cell population. **(H)** FACS analysis of TIL from control-EMT6 or HC-EMT6 tumors resected on day 14 (*n* = 6/group), specifically showing CD4+ and CD8+ regulatory T cell populations (CD25+FoxP3+). **(I)** FACS analysis of TIL from control-EMT6 or HC-EMT6 tumors resected on day 35 (*n* = 3/group), specifically showing CD4+ and CD8+ regulatory T cell populations (CD25+FoxP3+). ^*^P < 0.05.

Given that chemerin-overexpression failed to impact EMT6 proliferation *in vitro* or expression of MHC class I, CD1d, CD44, or PD-L1, we next wanted to study the impact of chemerin expression in the TME on *in vivo* growth. Using WT female BALB/c recipients, control or chemerin-expressing EMT6 tumor cells were orthotopically implanted into the mammary fat pad as described (22). To determine if the level of chemerin secretion from transduced clones affected *in vivo* tumor growth, we implanted low-chemerin (LC) and high-chemerin (HC)-secreting clones. The *in vivo* growth of HC EMT6 tumors was significantly suppressed compared to LC- or control-EMT6 cells (Figure 4A), with some mice showing complete suppression of *in vivo* tumorigenesis. To confirm this was not an effect of clonality, we utilized completely independent, bulk transduced EMT6 tumor cell lines (i.e., polyclonal) and saw a similar significant reduction in *in vivo* tumor growth (Figures 4A,B). This might suggest that an adequate concentration gradient of chemerin within the TME needs to be established to recruit anti-tumor leukocytes and suppress tumor growth. Indeed, there was an approximately 2-fold increase in the total CD45+ tumor infiltrating leukocytes (TIL) relative to tumor cells in HC-EMT6 tumors compared to LC- or control-EMT6 tumors at time of euthanasia (Figures 4C,D). We next looked at the composition of infiltrating leukocyte subsets in the TME by flow cytometry and identified significant increases in the relative percentages of total T cells, CD4+ T cells, and NK cells among total CD45+ cells in HC-EMT6 tumors compared with controls by day 35 of tumor growth (Figures 4E,F; Supplemental Figure 2). CD8+ T cells were also enriched among the total CD45+ cells in the HC-EMT6 tumors by day 35, compared to day 14 (Figure 4G). However, no significant differences in percentages of total T cells, CD4+ or CD8+ T cell subsets, B cells, NK cells, cDCs, pDCs, MDSCs, or macrophages among CD45+ TILs were detected between the two groups at an earlier time point in tumor growth (day 14, not shown), potentially suggesting that sufficient time is needed to establish an adequate concentration gradient of chemerin and resultant chemoattraction of effector cells. No significant differences were seen in either CD4+ or CD8+ regulatory T cells (CD25+FoxP3+) between the groups at either early or late time points (Figures 4H,I). Taken together, these data show high-chemerin expression within the EMT6 TME results in significant tumor growth suppression and a favorable anti-tumor skewing of both NK cells and T cells, as a percentage of total TIL.

### The Anti-Tumor Effects of Chemerin Are Mediated by NK Cells and T Cells

**Figure 5:**
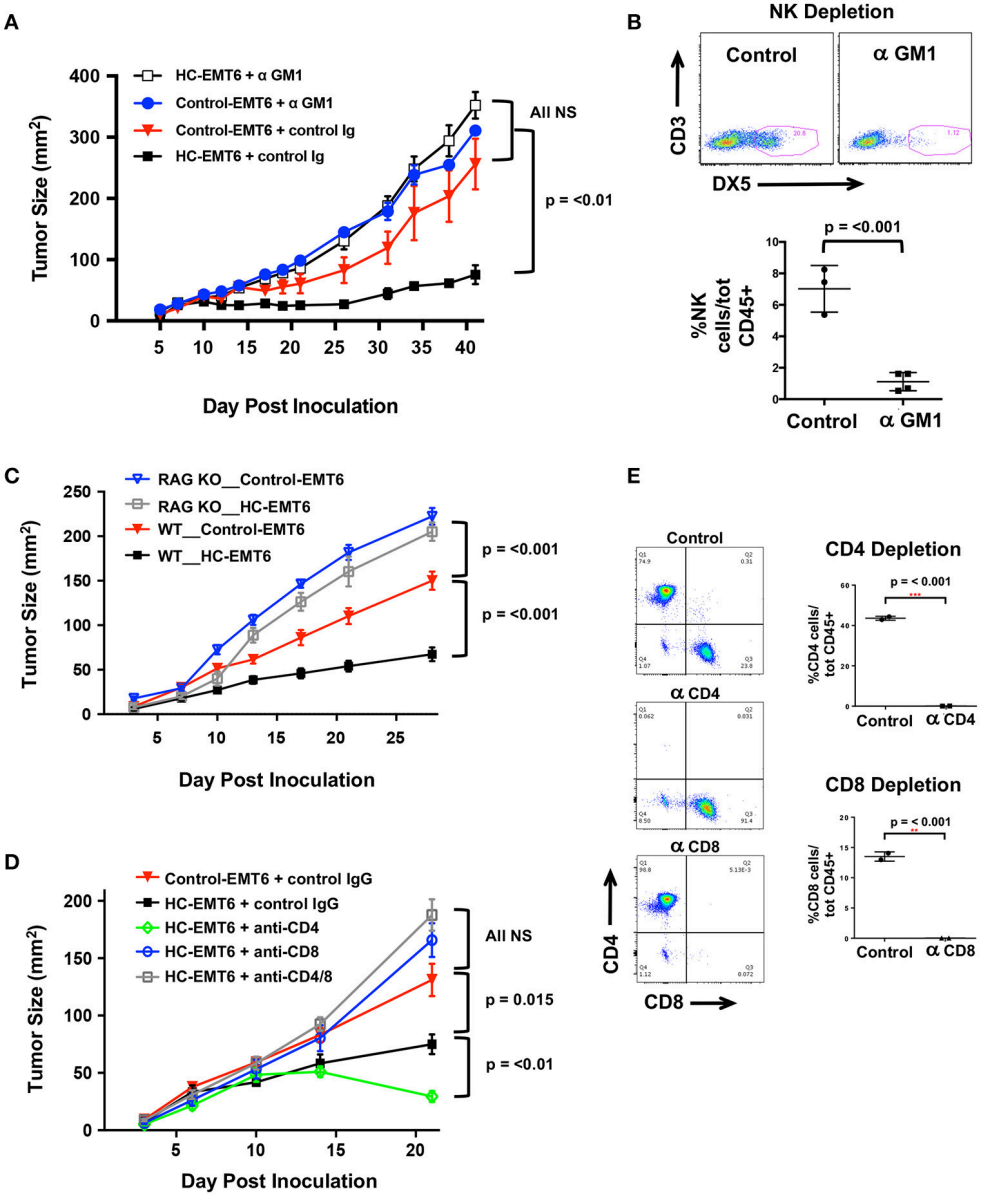
Depletion of NK, T cells abolishes chemerin-induced tumor suppression. **(A)** Control or high chemerin-expressing (HC) EMT6 cells were inoculated in wild type BALB/c mice (*n* = 4–5/group). NK cell depletion was accomplished using anti-asialo GM1 (control rabbit sera was used as a negative control) (Wako Chemicals). **(B)** Depletion of blood NK cells was confirmed in each experiment at either day −1 or day 0 (time of inoculation). **(C)** Control and chemerin-expressing lines were inoculated in both wild type (WT; *n* = 10/group) and Rag-1 knockout (RAG KO, Jackson Labs; *n* = 7/group). **(D)** Antibody depletion of T cell subsets (CD4, CD8) was accomplished using i.p., injection of 250 ug/500 ul PBS of anti-CD4 (clone GK1.5, BioXCell), anti-CD8β (Lyt 3.2) (clone 53-5.8, BioXCell) or both, or control rat IgG (Sigma). Control or depleting antibodies were used in both control and chemerin-expressing (HC) tumors as indicated (*n* = 5–6/group). **(E)** Depletion of blood T cell subsets was confirmed in each experiment at either day−1 or day 0 (time of inoculation). Graphs show mean/SEM from representative experiments with similar results (*n* = 4 experiments for NK depletion, *n* = 2 experiments each for T cell depletion, RAG KO studies). *P*-values are indicated from 2-tailed unpaired *t*-tests between indicated groups at time of euthanization.

Our initial *in vivo* EMT6 tumor data identified a correlation among high-chemerin expression by EMT6 tumors, increased NK cells and T cells in the TIL population and suppressed tumor growth. (Figure 4). To further define the cellular mechanism of action of chemerin-dependent tumor growth suppression, we selectively depleted candidate lymphocyte subsets (or used genetically-modified subset-deficient animals) and evaluated HC-EMT6 tumor growth. We first used anti-asialo GM1 to deplete NK cells. Control and chemerin-expressing lines were inoculated into mice treated with control sera or anti-asialo GM1 (Wako Chemicals) sera. Anti-asialo GM1 treatment had no effect on control EMT6 tumor growth *in vivo*, while similar treatment resulted in the complete abrogation of tumor suppression in the chemerin-expressing tumors (Figure 5A). There were no significant differences noted between the growth of control-EMT6 tumors (+/- anti-asialo GM1) and NK cell-depleted chemerin-expressing tumors (Figure 5A). The extent of NK cell depletion was confirmed by analysis of peripheral blood prior to tumor inoculation (Figure 5B; Supplemental Figure 3A). Next, to explore the potential role of adaptive immunity in chemerin-dependent EMT6-tumor growth suppression, we used Rag1 KO mice, which lack mature T and B cells (35). Growth suppression by tumor-secreted chemerin was only seen in wild type mice and was completely abrogated in RAG KO mice (Figure 5C), suggesting a requirement of the adaptive immune response in this model. Given the lack of change in B cells and the significant increase in T cells in the TIL population in chemerin-expressing tumors, we then set out to define specific T cell subsets responsible for the chemerin-dependent anti-tumor effect. We used specific antibodies to deplete CD4+ and/or CD8+ T cells as indicated. Control antibody treatment did not affect suppression of tumor growth in chemerin-expressing tumors. However, depletion of CD8+ T cells in chemerin-expressing tumors—either alone or in combination with CD4+ T cell depletion—resulted in growth comparable to control tumors (Figure 5D). T cell subset depletion was confirmed by analysis of peripheral blood, which was essentially complete (Figure 5E; Supplemental Figure 3B). Interestingly, CD4+ T cell depletion alone in chemerin-expressing tumors resulted in improved tumor growth suppression (Figure 5D). Recently published data show that CD4+ T cell depletion in the EMT6 model results in a significant increase in CD45+ TIL, with a ~3-fold increase in IFNγ+CD8+ T cells in the draining lymph nodes compared to controls. CD4+ T cell depletion—as in our model—resulted in significantly reduced tumor growth, hypothesized to be due to a reduction in immunosuppressive regulatory CD4+ T cells (40). In line with this data, analysis of our control and T cell depleted cohorts showed a significant increase (~3-fold) in total CD45+ TIL only in the CD4+ T cell depleted mice (not shown). Taken together, these data suggest critical roles for both NK and CD8+ T cells in mediating chemerin tumor suppression.

## Discussion

**Figure 6:**
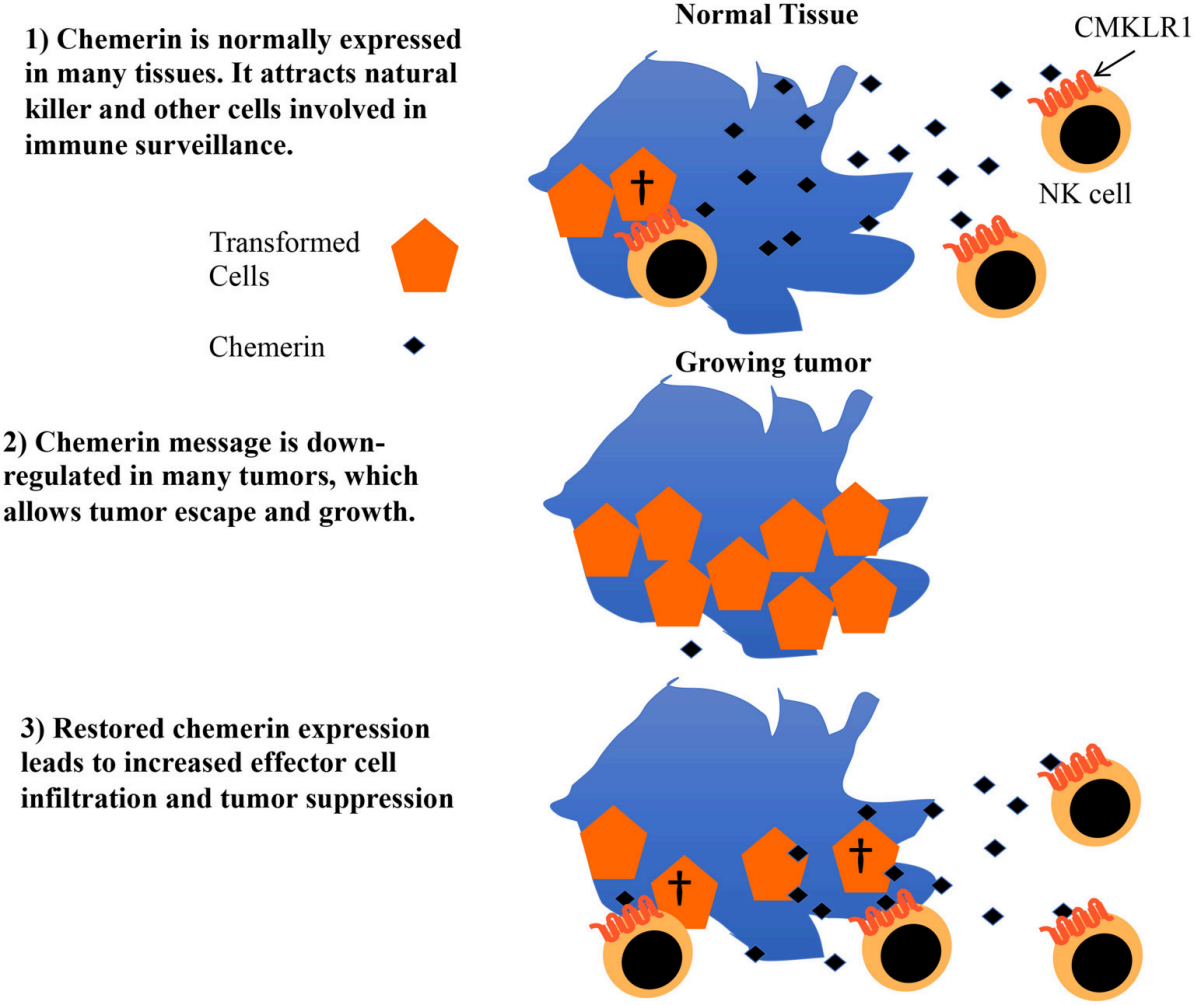
Proposed model for role of chemerin in tumor immune surveillance and suppression. Chemerin/RARRES2 is down-regulated in breast and other tumors compared to their normal tissue counterparts. Data from our mouse tumor models show that forced over-expression of chemerin within the tumor microenvironment (TME) results in an increase in tumor-infiltrating leukocyte effector subsets (NK cells, CD8+ T cells) that are required to suppress tumor growth. Strategies focused on augmenting chemerin in the TME may represent an attractive strategy to increase effector cell composition within the tumor and potentially favorably impact the effect of existing immunotherapies such as checkpoint inhibitors in otherwise treatment-refractory tumors.

Chemerin is a multifunctional protein with wide tissue expression and myriad roles in host defense, implicated in antibiosis, angiogenesis, as well as chemoattraction of leukocytes (43). Several groups have described its dysregulation in the context of tumorigenesis, with the majority—but not all—showing decreased chemerin/*RARRES2* expression within malignant tissues (20–22, 42, 44–48). Our group was the first to show tumor suppression via therapeutic modulation of chemerin in a mouse tumor model, with now several studies confirming the role of chemerin as a tumor suppressor in various settings (22, 45, 46, 48, 49). Importantly, two independent studies showed not only improved patient survival but also increased immune effector cell infiltrates in tumor samples with higher chemerin expression (20, 21). Our prior studies in the B16F0 mouse melanoma model showed increases in tumor-infiltrating NK and T cells with forced overexpression of chemerin by tumor cells, with suppression mediated by NK cells in that model (22). This led us to hypothesize that chemerin may play a key role in tumor immune surveillance and, further, that malignant tissues may selectively down-regulate chemerin/*RARRES2* as a means of immune escape (Figure 6).

Human breast cancers have variable levels of infiltrating immune cells, with ER/PR+HER2- subtypes typically showing the lowest (2). Breast cancer subtypes with high TILs may also show higher expression of checkpoint molecules such as PD-1 and CTLA-4 (50), which may play a role in higher response rates to checkpoint inhibitors in these tumor subtypes (e.g., ER/PR-HER2-, HER2+) (51). Decreased levels of TIL have been described in metastatic breast tumors compared to matched primary tumors (52), suggesting a role for immune escape in breast cancer progression. Thus, strategies to increase TIL and improve immunosurveillance in breast cancer are attractive from a therapeutic standpoint.

Here, we present—for the first time to our knowledge—studies focused on the expression and role of chemerin/*RARRES2* in human breast tissues and a mouse model of breast cancer. Using the fully immune competent mouse EMT6 breast tumor model, we have shown that overexpression and secretion of chemerin by tumor cells significantly suppressed tumor growth *in vivo*. As in our melanoma model, chemerin appears to have no significant effect on tumor intrinsic proliferation or phenotype *in vitro*, though this may be a function of specific tumor types as well as the presence or absence of chemerin receptors on tumor cells, as others have shown direct effects of chemerin on tumor cells (45, 46, 48, 49). *RARRES2*-transduced EMT6 clones with lower expression of chemerin grew similarly to control cells *in vivo*, suggesting that in this model adequate expression and secretion of chemerin within the TME is necessary to successfully establish the concentration gradient necessary to recruit leukocytes.

The EMT6 mouse tumor model has recently been shown to recapitulate an “immune excluded” tumor phenotype with exclusion of CD8+ T cells from the tumor parenchyma, often seen in human tumors such as urothelial cancers (31). Importantly, we found meaningful increases in both NK and T cells within chemerin-expressing tumors compared to controls, similar to our findings in the melanoma model. Depletion studies indicate important roles for NK and CD8+ T cells in mediating the tumor suppressive effects of chemerin in this model, not surprisingly as supportive roles of NK cells in T cell function and the adaptive immune response are well described (53–56). Though chemerin does not seem to directly recruit CD8+ T cells via CMKLR1 interactions in this model, there is compelling evidence in the literature to suggest that NK cells mediate various functions that enhance CD8+ T cell cytolytic activity; for example, NK cells have been shown to moderate CD8+ T cell priming during influenza A viral infection and activate CD8+ T cell anti-tumor activity in the YAC-1 mouse lymphoma model (57, 58). Other studies have found that intratumor NK cell recruitment induces further leukocyte infiltration into the tumor (59), together articulating the point that chemerin may not need to act directly on CD8+ T cells to play a role in chemerin-dependent tumor growth inhibition. Additionally, ongoing studies include the impact of chemerin expression on the establishment of immune memory as well as the development of metastatic disease in this model.

Our *de novo* studies of human breast tissues using two independent cohorts of normal, IDC, and ILC samples across two assay platforms confirm large publicly available microarray datasets showing RARRES2 is significantly down-regulated in breast malignancies. Additionally, analysis of two mRNA microarray datasets showed that reduced chemerin levels significantly correlated with poor survival outcomes. In our *in vivo* experiments, we did not directly assess the effects of chemerin down-regulation/silencing during tumorigenesis in the EMT6 model. Rather, we focused on studying the potential therapeutic activity of restoring and/or overexpressing chemerin in the TME. Additional tumor studies are needed in animals with spontaneous carcinomas to determine whether chemerin down-regulation in the TME correlates with poor survival and thus models the clinical results we described in Figures 1, 2. Given the variability within and across tumor types, evaluation of chemerin/RARRES2 and receptor expression will be important prior to pursuing human translational studies. Importantly, recently published data provides a mechanistic link between chemerin and PTEN expression and function in hepatocellular carcinoma (48), suggesting chemerin may have other tumor suppressive mechanisms of action in addition to the recruitment of immune effector cells into the TME. Taken together our data elucidate mechanistic insights into the role of chemerin in breast tumor suppression and provide rationale for translational studies in human breast cancer.

## Ethics Statement

This study was carried out in compliance with the ethics policies of Washington University School of Medicine. All animal studies were carried out under institutional IACUC-approved protocol, and human tissues were obtained from the St. Louis Breast Tissue Registry (funded by The Department of Surgery at Washington University School of Medicine, St. Louis, MO) in accordance with the guidelines established by the Washington University Institutional Review Board (IRB #201102394) and WAIVER of Elements of Consent per 45 CFR 46.116 (d). All patient information was de-identified prior to sharing with investigators. All of the human research activities and all activities of the IRBs designated in the Washington University (WU) Federal Wide Assurance (FWA), regardless of sponsorship, are guided by the ethical principles in The Belmont Report: Ethical Principles and Guidelines for the Protection of Human Subjects Research of the National Commission for the Protection of Human Subjects of Biomedical and Behavioral Research.

## Author Contributions

RP, EB, and BZ conceived and designed experiments. RP, W-IL, PW, GV, KR, JR, YZ, LN, and NS performed experiments. RP, PW, GV, KR, WS, VN, and BZ analyzed data. RP, PW, WS, GV, KR, and BZ wrote the manuscript.

### Conflict of Interest Statement

W-IL is currently employed by Dynavax Technologies, Berkeley, CA; no experiments, data-sharing or analyses, or other work were performed at or in collaboration with Dynavax. The remaining authors declare that the research was conducted in the absence of any commercial or financial relationships that could be construed as a potential conflict of interest.
