# Supplementary figures and images for: Chemerin Suppresses Breast Cancer Growth by Recruiting Immune Effector Cells Into the Tumor Microenvironment
Source: Front Immunol. 2019 May 8;10:983. doi: 10.3389/fimmu.2019.00983 (PMC6518384; doi:10.3389/fimmu.2019.00983)

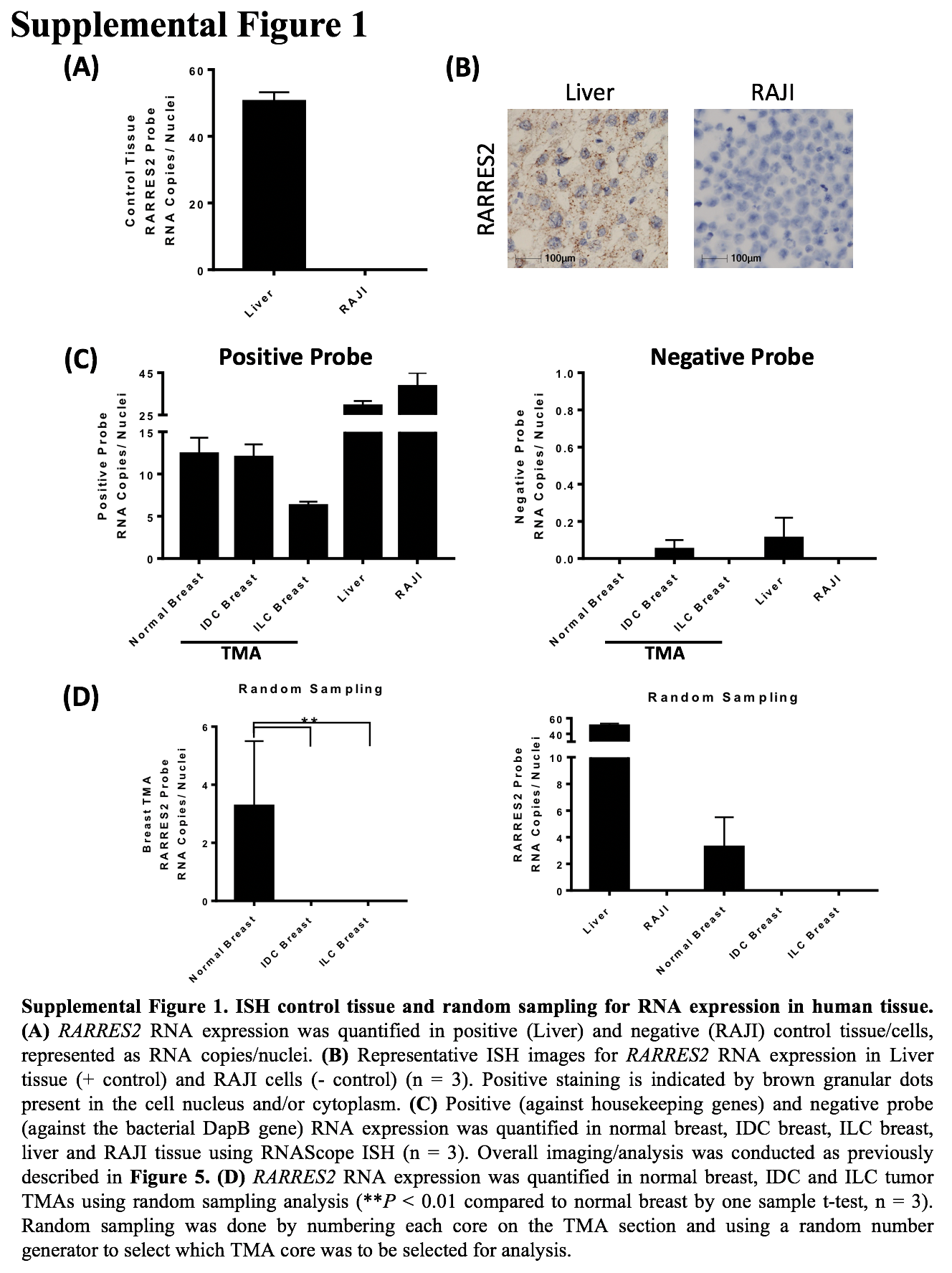

Supplement: Supplementary file 1 [file Image_1.JPEG]

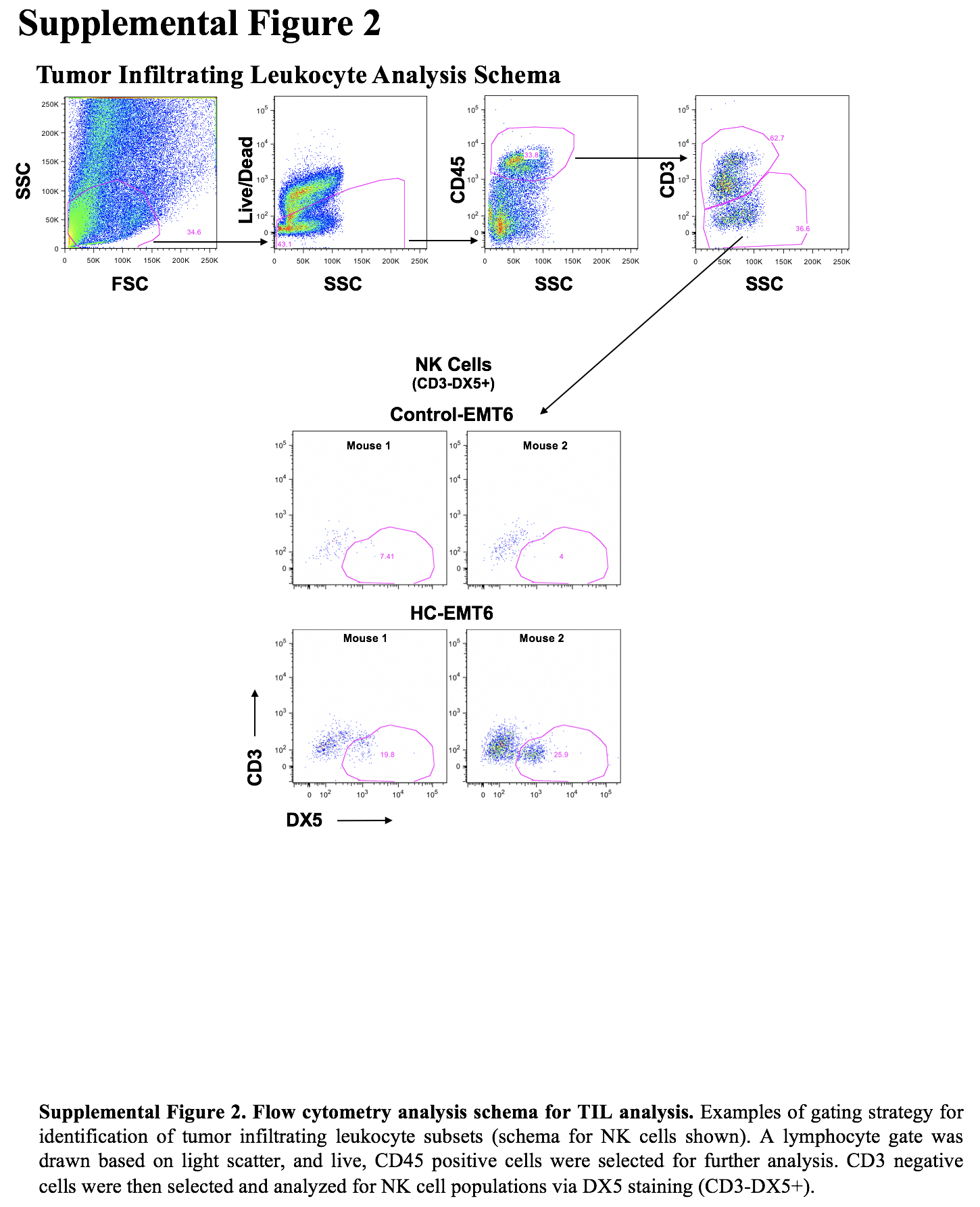

Supplement: Supplementary file 2 [file Image_2.JPEG]

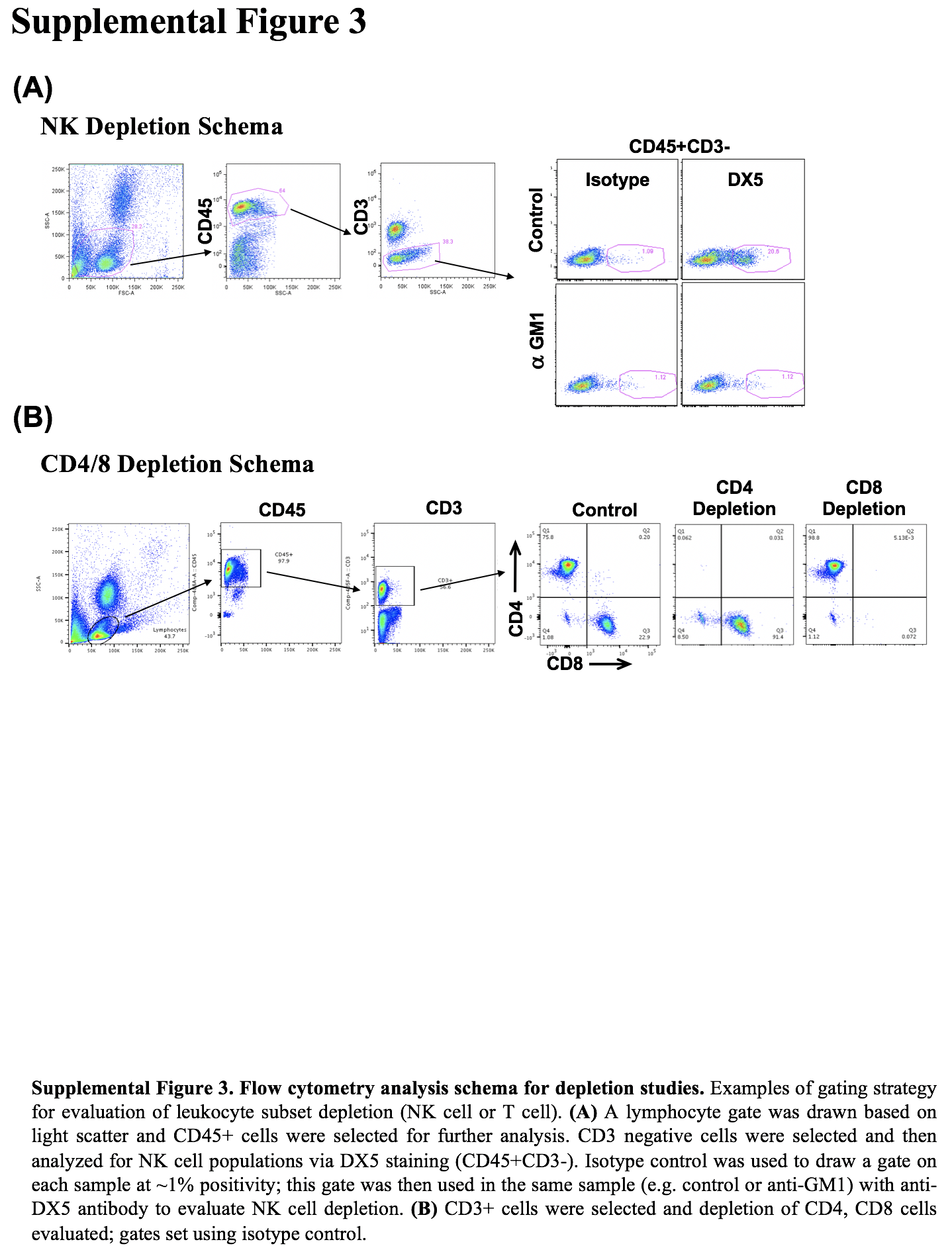

Supplement: Supplementary file 3 [file Image_3.JPEG]
